# Comparative Bioactive Compounds and Mineral Properties of South African and Lesotho *Artemisia afra* (Jacq.) Genotypes

**DOI:** 10.3390/plants13081126

**Published:** 2024-04-17

**Authors:** Matumelo Rafiri, Moosa Mahmood Sedibe, Goitsemang Mahlomola Hendry Dikane

**Affiliations:** Department of Agriculture, Central University of Technology, Free State, Private Bag x20539, Bloemfontein 9301, South Africa; marafiri@yahoo.co.uk (M.R.); gdikane@cut.ac.za (G.M.H.D.)

**Keywords:** ethnomedicine, ICP-OES (inductive coupled plasma optical emission spectrometric), indigenous knowledge, Keller–Kiliani, Lengana, medicinal plants, PCA (principal component analysis), phytochemicals, Salkowski, umhlonyane

## Abstract

*Artemisia afra* is a plant that grows in the northern, central, and coastal regions of South Africa, as well as in neighboring countries such as Eswatini and Lesotho. These phytochemicals can be used as active compounds in plant-based medicine. Therefore, it is important to determine how plant minerals and phytochemicals, particularly bioactive compounds, are affected by the geolocation in which they grow. This study aimed to evaluate the mineral and phytochemical properties of *A. afra* genotypes in the southern regions of Africa. Leaf samples of *A. afra* genotypes were collected from Lesotho, in Mohale’s Hoek and Roma. In South Africa, leaf samples were collected in Wepener and Hobhouse, and 80 plants were randomly selected for phytochemical and mineral analyses. This study reveals that phosphorus, calcium, potassium, iron, and zinc loaded positively to the first principal component, while copper loaded positively to the second principal component with variabilities of 29.95% and 21.12%, respectively. Furthermore, both the Mohale’s Hoek and Hobhouse genotypes exhibited relatively high levels of ascorbic acid, phenolic compounds, flavonoids, and tannins. It is worth noting that genotypes from Roma and Wepener showed higher levels of foliar magnesium. Thus, the Mohale’s Hoek and Hobhouse genotypes could be recommended for their better phytochemical contents.

## 1. Introduction

Medicinal plants produce phytochemicals that protect against injuries, diseases, and pest attacks. Phytochemicals can be used as active compounds in plant-based medicine. Tribal medicine accounts for 40% of the total consumption of medicinal products [1]. These medicinal plants are used in both developed and developing countries, whether directly as indigenous medicine or indirectly as pharmaceutical products.

According to [2], up to 80% of the global population rely on medicinal plants as a primary source of medicine for their health care. Most of these plants grow around homesteads in Africa and have abundant antioxidants [2]. Medicinal plants also grow freely in African homesteads, and they have versatile bioactive compounds for human and livestock welfare. These medicinal plants are also used in essential oils, food, and animal supplements [2].

According to [3], phenolic compounds are secondary metabolites that are produced by plants through the shikimate, pentose phosphate, and phenylpropanoid pathways. These compounds consist of benzene rings with one or more hydroxyl substituents, and they range in complexity from simple phenolic molecules to highly polymerized compounds. Plants and plant-derived products have been used for centuries to maintain human health and improve quality of life. Many studies have highlighted the potential health benefits of plant polyphenolics, which are known for their strong antioxidant properties. These compounds have been scientifically proven to prevent various oxidative stress-related and chronic diseases, including cancer, cardiovascular disease, and neurodegenerative disorders. Despite their prevalence in the plant kingdom, researchers have only recently started to focus on the health benefits of phenolics [4].

The Artemisia genus is known for treating a wide range of human illnesses, such as fever, skin infections, rheumatism, snakebites, jaundice, dysentery, malaria, toothache, hyperthermia, headaches, dyspepsia, colic, diabetes, and bladder and kidney infections. Furthermore, *Artemisia absinthium* (L.) has been used as an antispasmodic, stomachic, cardiac stimulant, and anthelmintic and for memory restoration in cases of failing mental function [5,6,7].

*A. afra* is a plant that grows in various geolocations of South Africa, including neighboring countries such as Eswatini and Lesotho. The plant is commonly known as Umhlonyane and Lengana by the indigenous Nguni and Bakone people. However, there is limited scientific information available about the mineral and phytochemical composition of South African *A. afra* genotypes. Therefore, investigating how phytochemicals, such as bioactive compounds and minerals, are influenced by the geolocations in which they grow is crucial. The goal of this study is to evaluate the phytochemical and mineral properties of *A. afra* genotypes in different regions of southern Africa.

## 2. Results

### 2.1. Phytochemicals

#### 2.1.1. Qualitative Analysis

Phytochemical screening of the crude methanolic *A. afra* extracts revealed the presence of flavonoids, tannins, phenolics, terpenoids, steroids, saponins, and alkaloids in all the genotypes tested. The presence of phytochemicals is indicated as slightly present (+), moderately present (++), highly present (+++), and not detected (−) (Table 1). Tannin content was moderately present in the Wepener-WE, Mohale’s Hoek-MH, and Roma-RO genotypes, while, in the Hobhouse-HO genotypes, tannin was only slightly present. Terpenoid was moderately present in the WE, HO, and RO genotypes, and MH terpenoid was slightly present. Steroids were highly present in genotypes collected from MH, RO, and WE, with only HO steroids being slightly present. Saponins were slightly present in all genotypes collected in all the locations of Lesotho and South Africa.

#### 2.1.2. Quantitative Analysis

**Figure 1:**
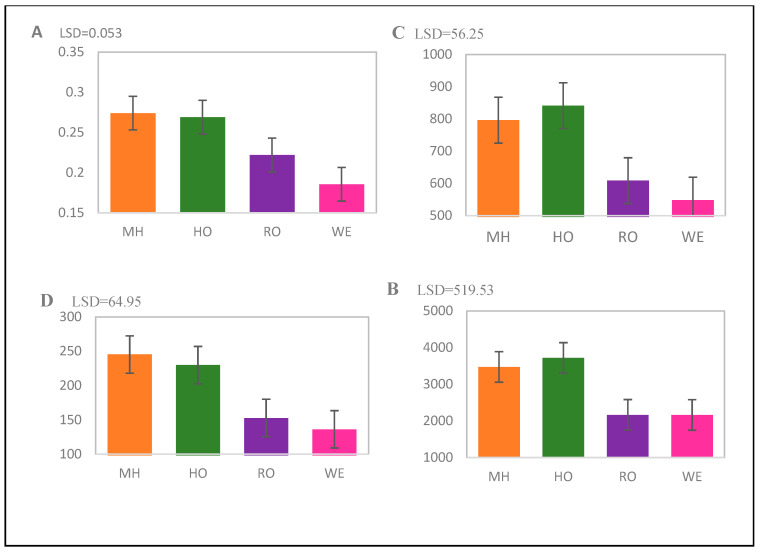
(**A**) ascorbic, (**B**) phenolic, (**C**) flavonoid, and (**D**) tannin contents of *A. afra* genotypes from four geolocations: Lesotho (Mohale’s Hoek (MH): 30.1426° S, 27.4674° E, and Roma (RO): 29.5212° S, 27.7755° E) and South Africa (Wepener (WE): 29.7294° S, 27.0206° E, and Hobhouse (HP): 29.5258° S, 27.1452° E).

An analysis of variance revealed the significant effect of geolocation on the contents of ascorbic acid (*p* < 0.01) and flavonoid (*p* < 0.001) in *A. afra* genotypes. As depicted in Figure 1, genotypes collected from MH and HP had high levels of ascorbic acids and flavonoid contents. The tannin and phenolic contents showed a similar trend where the geolocation significantly affected these compounds at a *p*-value of *p* < 0.001 for each compound (Figure 1).

### 2.2. Mineral Contents

#### 2.2.1. Foliar Mineral Contents

Table 2 shows a significant difference in foliar mineral content occurring between locations for nitrogen (*p* < 0.001), phosphorus (*p* < 0.05), calcium (*p* < 0.0001), magnesium (*p* < 0.0001), and manganese (*p* < 0.05). Increased foliar nitrogen was observed in genotypes collected from MH, followed by RO. For MH and WE, more phosphorus was recorded on *A. afra* foliage than for HP and RO. However, foliar Ca was increased in genotypes collected from HP, MH, and WE. Foliar Mg was elevated in genotypes collected from RO and WE. For MH, RO, and WE, high levels of manganese were recorded. No significant differences occurred between locations for the remaining foliar minerals (i.e., K, Na, Fe, Cu, and Zn).

#### 2.2.2. Principal Component Analysis (PCA)

**Figure 2:**
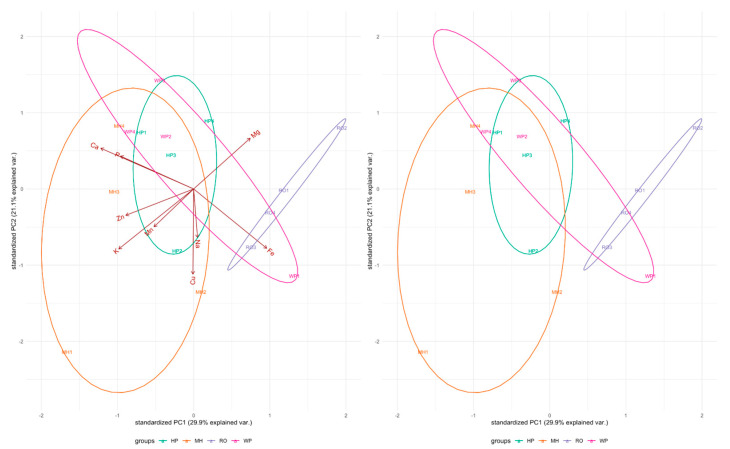
Foliar mineral content variability principal component analysis of *A. afra* genotypes from four geolocations: Lesotho (Mohale’s Hoek (MH): 30.1426° S, 27.4674° E, and Roma (RO): 29.5212° S, 27.7755° E) and South Africa (Wepener (WE): 29.7294° S, 27.0206° E, and Hobhouse (HP): 29.5258° S, 27.1452° E).

**Figure 3:**
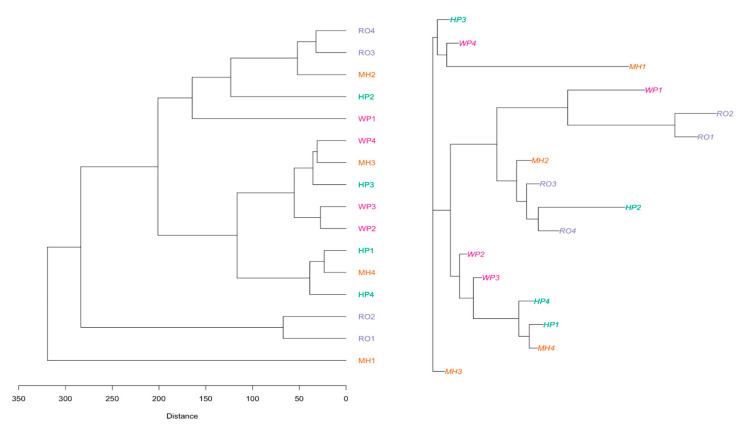
Foliar mineral relationships between populations of *A. afra* using unweighted pair group method with arithmetic mean and neighbor-joining method. Lesotho (Mohale’s Hoek (MH): 30.1426° S, 27.4674° E, and Roma (RO): 29.5212° S, 27.7755° E) and South Africa (Wepener (WE): 29.7294° S, 27.0206° E, and Hobhouse (HP): 29.5258° S, 27.1452° E).

The PCA loading for mineral data is shown in Figure 2. Phosphorus, Ca, K, Fe, and Zn loaded positively to the first PCA, while Cu loaded positively to the second PCA with variabilities of 29.95% and 21.12%, respectively. The foliar mineral relationship between populations of *A. afra* using UPGMA and neighbor-joining methods is shown in Figure 3. For genotypes collected in RO and MH, three distinct groupings were discovered. The neighbor-joining (NJ) method was applied for cluster development, using foliar minerals to construct the neighbor-joining tree. Three major clusters were formed (Figure 3): cluster I contained only one genotype collected from MH; cluster II contained 12 genotypes collected from MH, HP, WE, and RO; and cluster III contained only three genotypes collected from MH, WP, and HP.

## 3. Discussion

The phytochemical screening of the crude extracts revealed the presence of some secondary metabolites such as terpenoids, alkaloids, and tannins. Previous research has shown that terpenoids, including triterpenes, sesquiterpenes, and diterpenes, possess antibiotic, insecticidal, anthelmintic, and antiseptic properties [8,9,10,11]. All the plant extracts contained terpenoids and glycosides. Ref. [8] reported the presence of tannins in the water and ethanolic extracts of the plant samples. Alkaloids were only detected in the ethanolic extract from South Africa and Kenya. The results also showed that ethanol and water solvents extracted more phytochemicals due to their high polarity. Flavonoids are natural chemicals that can be found in various fruits and vegetables, as well as in tea, wine, nuts, seeds, herbs, spices, chocolate, and legumes. These chemicals play an essential role in growth and plaque protection in vegetables, as stated by [12]. Flavonoids have numerous human health benefits, such as antioxidant properties, anti-inflammatory properties, gene mutation prevention, and cancer growth prevention. Additionally, they have a positive impact on the functioning of cellular enzymes, which are responsible for triggering chemical processes in cells. According to [13], consuming foods that are rich in flavonoids may be an effective way to manage hypertension. Flavonoids in *A. annua* leaves can inhibit the CYP450 enzymes that affect artemisinin absorption and metabolism. These compounds increase under abiotic stress by enhancing the phenylpropanoid biosynthetic pathway [14,15,16]. According to this study, *A. afra* samples from MH and HP locations had the highest flavonoid levels at 796 and 841, respectively. In this study, the highest flavonoid levels, of up to 796 and 841, were recorded in *A. afra* obtained in MH and HP, respectively. In contrast, flavonoid levels in Burundi, Tanzania, Senegal, South Africa, and Kenya were recorded as 242, 165, 162, 140, and 139, respectively [8]. However, the study did not specify from which geolocation the *A. afra* material was collected [8].

Tannin plant extracts are used in ruminant diets to increase meat and milk quality and oxidative stability [17]. Tannins also affect various physiological processes in humans, such as accelerating blood coagulation, regulating serum cholesterol levels to prevent liver complications, and regulating immune responses [18]. Tannin concentrations in plants change when ecological factors, such as soil nutrients, photoperiod, climatic temperatures, carbon dioxide, and water sources, are negatively changed [19]. In Iran, *Artemisia annua* leaves contained 340 mg of tannins/100 g, but the stems had just 30 mg/100 g [20]. A Turkish study discovered an average of 420 mg of tannins/100 g in *Artemisia absinthium* [21]. In our study, the highest tannin levels, of up to 245 and 229, were recorded in *A. afra* obtained in MH and HP, respectively; in these respective geolocations, high levels of phenolic contents were also recorded. As per the findings of this study, it can be inferred that the presence of polyphenolic components in *A. afra* plays a vital role in its pharmacological effects [8].

MH and HP had the highest levels of ascorbic acid. The amount of vitamin C present in plants is affected by their growth stage and changes in their environment [22,23]. Light is an important factor in regulating vitamin C levels [24,25]. Vitamin C plays a vital role in the proper functioning of photosynthesis in chloroplasts. Ascorbic acid is indispensable for both plants and animals. This vital nutrient plays a crucial role in maintaining the delicate balance of redox reactions and acts as a cofactor for enzymes that regulate essential biological processes such as photosynthesis, hormone biosynthesis, and antioxidant regeneration. In animals, ascorbate is involved in the synthesis of carnitine and collagen, an important component of skin, scar tissue, tendons, ligaments, and blood vessels [26,27].

Plants contain varying minerals and bioactive compounds, with some being essential for the regulation of human metabolism in ethnomedicine, as reported by [28,29]. Moreover, the study found that minerals act as cofactors for enzymes that mediate important human biochemical reactions, and their concentrations may indicate heterozygosity among genotypes. Soils act as reservoirs for minerals and provide plants with the vital minerals they need for growth. Therefore, a shortage of these minerals in the soil can cause stress for the plant, leading to the production of complementary substances for survival under stress conditions, as reported by [30]. Mineral availability often influences the population distribution of plants. Soil properties and environmental factors affect soil functions, such as drainage, mineral availability, transportation, and uptake by plants for growth and development, as reported by [31,32]. The authors of [33] reported that some minerals are absorbed more efficiently than others, with higher concentrations in cell sap than in the external soil solution and that there are distinct differences between them.

Differences can occur between plant species and within species, and this was observed in *A. afra*. For example, soil collected from WE had high concentrations of P, Mg, Fe, and Mn, and foliar mineral analysis showed significantly higher levels of these minerals in WE compared to other locations. On the other hand, the RO soil had a better amount of Zn, and this mineral was not affected by location. The highest levels of Ca and Cu were found in the MH soil, while mineral analysis only agreed on foliar Ca. In addition, foliar N was relatively low despite high soil N, most likely due to the N dynamics being affected by the stabilization of the soil system during the ammonification phase of N mineralization, as opposed to destabilization during nitrification. The authors of [34] found that this stabilization can lead to some benefits, such as reduced N losses through leaching and denitrification, as well as energy savings for the plant. A distinct observation was made in terms of the high foliar magnesium scores in the distant geolocations of WE and RO. Moreover, further studies will have to be conducted to explain this observation. In [35], where the feasibility of using DArTseq-derived single-nucleotide polymorphisms (SNPs) was used to predict the spatial distribution of *A. afra* gene diversity, the authors arrived at a similar conclusion to that of our mineral analyses, in that, per locality, low subspecies genetic diversities and uniformities were found. However, *A. afra* genotypes collected from MH and HP had relatively high contents of ascorbic acid, phenolic compounds, flavonoids, and tannins. Among all the foliar minerals evaluated, only foliar Mg showed a similar trend to the phytochemicals.

## 4. Materials and Methods

### 4.1. Research Site Description

The modified Whittaker nested vegetation sampling method was used to collect 80 leaf samples from four different locations in Lesotho, including Mohale’s Hoek (located at 30.1426° S, 27.4674° E) and Roma (located at 29.5212° S, 27.7755° E). In South Africa, leaf samples were collected in Wepener (located at 29.7294° S, 27.0206° E) and Hobhouse (located at 29.5258° S, 27.1452° E). This was carried out systematically by placing ten 0.5 × 2 m (1 m^2^) subplots along the inside border, two 2 × 5 m subplots in alternative corners, and a 5 × 20 m subplot at the center of the plot. The subplots were used to collect plants and surrounding soil from a depth of 60 cm [36]. The soil chemical analysis results are presented in Table 3.

### 4.2. Sample Preparation and Extraction

The leaves were cleaned with purified water and air-dried in a fume hood over seven days. The dried leaves were pulverized, packed in zip-up plastic bags, and stored in a fridge (4 °C) for subsequent phytochemical extraction. About 2 g of the substance was weighed into a clean, dry, 50 mL Erlenmeyer flask; 20 mL of methanol was added, and the flask was ultrasonically heated at 50 °C for about 30 min. After this, the mixture was filtered through Whatman No. 1 filter paper into an amber bottle. The process was repeated, and the extracts were combined and stored in the fridge for phytochemical analyses.

#### 4.2.1. Chemicals and Standards Used

The chemicals used in this study—2,2-diphenyl-1-picryl-hydrezyl (DPPH), 2 N Folin-Ciocalteu reagent, polyvinyl polypyrolidone (PVPP), tannic acid, gallic acid, quercetin hydrate, rutin, Tocopherol standard solution, and Fehling’s solution—were obtained from Sigma Aldrich. All the chemicals were analytical grade, and no further purification of the chemicals was conducted. 

#### 4.2.2. Phytochemical Screening Tests

Following the standard protocols described by [37,38,39], with some modifications, a qualitative phytochemical screening of the *A. afra* methanolic extracts was performed to identify the presence of the following groups of compounds: alkaloids, flavonoids, phenolics, steroids, tannins, and terpenoids.

#### 4.2.3. Flavonoids

Sodium hydroxide test: To 0.5 mL of methanolic extract, about 0.5 mL of 2.0% NaOH was added, and the solution turned an intense yellow color. The addition of a few drops of diluted HCl acid, which turned the yellow solution colorless, indicated the presence of flavonoids. Ferric chloride test: To 0.5 mL of methanolic extract, a few drops of 10% ferric chloride solution was added. The formation of a green-blue solution indicated the presence of a phenolic hydroxyl group. About 10 mL of stock solution was diluted to 50 mL with distilled water in a standard flask to produce a working standard solution of quercetin with a concentration of 200 μg/mL

#### 4.2.4. Tannins

The addition of three drops of 2% Ferric chloride solution to 1 mL of methanolic extract produced a blue-black solution, indicating the presence of tannins.

#### 4.2.5. Phenols

The addition of two drops of 5% Ferric chloride to 1 mL of methanolic extract produced a dark blue solution. The color change indicated the presence of phenolic compounds. About 5 mL of stock standard solution was diluted to 100 mL with distilled water in a standard flask to obtain a working standard solution with a concentration of 50 μg/mL of gallic acid.

#### 4.2.6. Terpenoids

To methanolic extract (1 mL), 1 mL of chloroform was added, and the mixture was shaken well. To this mixture, 1 mL of acetic anhydride was added, followed by the addition of 1 mL of H_2_SO_4_. The formation of a reddish-violet color indicated the presence of triterpenoids.

#### 4.2.7. Steroids

An ethanolic extract of the sample (1 mL) was added to a test tube. To the sample extract, 1 mL of acetic anhydride was added, followed by 1 mL of concentrated H_2_SO_4_. The solution changed color from violet to blue, indicating the presence of steroids. 

#### 4.2.8. Saponins

A methanolic extract from the sample (1 mL) was added to 3 mL of distilled water; this mixture was shaken vigorously for about 1 min. The formation of an intense froth, which persisted for 1 h, indicated the presence of saponins in the sample.

#### 4.2.9. Alkaloids

Wagner’s test: About 2 mL of distilled water was added to 1 mL of the methanolic extract of the sample and acidified with five drops of 1.5% HCl, followed by the addition of five drops of Wagner’s reagent. A strong brown precipitate formed, indicating the presence of alkaloids in the sample.

### 4.3. Quantitative Analysis

Using UV/V spectrometry techniques, a quantitative phytochemical evaluation of the *A. afra* extract samples concerns the determination of the concentration of phenolic acids, flavonoids, and tannins in the sample materials. All analyses were carried out in triplicate.

#### 4.3.1. Total Flavonoids Content

For the preparation of quercetin standard stock solution concentrate, 1000 µg/mL was prepared by weighing 100 mg of quercetin into a 100 mL volumetric flask and filling it to the 100 mL mark with distilled water. Into a series of five 50 mL volumetric flasks, 0.5, 2.5, 5, 10, and 20 mL of the quercetin stock solution were pipetted and filled with distilled water to the mark. Then, 4% sodium hydroxide and 10% aluminum chloride solutions were prepared, and 2 mL of each was added to each of the five volumetric flasks. The volumetric flask mixture was incubated for 15 min after the mixture was allowed to cool at room temperature. The standard solution was run using a UV/V spectrophotometer at 510 nm wavelength to obtain the Standard Calibration Curve for total flavonoid content determination.

#### 4.3.2. Total Ascorbic Acid (Vitamin C) Content

An ascorbic acid working standard solution of 100 ppm concentration was prepared by weighing 25 mg ascorbic acid into a 25 mL volumetric flask and diluting with 0.4% oxalic acid up to the 25 mL mark. Respectively, into a series of five 25 mL volumetric flasks (S1–S5), 0.5, 0.75, 1, 1.25, and 1.5 mL of the stock solution were pipetted. To each of the solutions (S1 to S2) 5 mL of 10% H_2_SO_4_ was added, and these were then filled with 5% ammonium molybdate to the 25 mL mark. Then, the flasks were incubated for 30 min and the absorbance was measured at wavelength 494 nm to obtain the ascorbic acid calibration curve.

#### 4.3.3. Total Phenolic Content

A standard stock solution (gallic acid) was prepared by weighing 50 mg gallic acid into a 50 mL volumetric flask and diluting it with methanol to the 50 mL mark. A working standard gallic acid solution of concentration 50 µg/mL was prepared by diluting 5 mL of the stock solution to 100 mL with distilled water. The Folin–Ciocalteu reagent concentration was prepared by diluting 2 mL of the reagent with 2 mL of distilled water. Into five 50 mL volumetric flasks, each containing 0.2, 0.4, 0.6, 0.8, and 1 mL of the working standard, 5% sodium carbonate was added. This was followed by the addition of 0.5 mL of the diluted Folin–Ciocalteu reagent, and the mixture was incubated for 40 min in the dark. The absorbance of the deep blue mixture was measured against the blank (distilled water) at 725 nm using a spectrophotometer.

#### 4.3.4. Tannin Content

The tannic acid standard stock solution was prepared by weighing 50 mg of tannic acid into a 50 mL volumetric flask and diluting it with methanol to the 50 mL mark. The working standard solution of a concentration of 50 µg/mL of tannic acid was prepared by diluting 5 mL of the tannic acid standard stock solution to the 100 mL mark of the volumetric flask with distilled water. Into five separate 50 mL volumetric flasks, 1, 2, 3, 4, and 5 mL of the working solution were pipetted. To the flasks, 1 mL of 5% sodium carbonate and 0.5 mL of Folin–Ciocalteu (1N) were added; these were shaken to mix well and allowed to stand for 30 min. The absorbance was determined using UV/V at a wavelength of 700 nm against the blank (distilled water).

### 4.4. Mineral Content Determination

Oven-dried leaves of *A. afra* were used to determine (N, P, K, Ca, Mg, S, Fe, Zn, and Cu) mineral contents using a Dumas combustion method [33,40,41] and a Leco FP-528 combustion nitrogen analyzer (LecoCorp. St. Joseph, MI, USA). An automated sample loader (CNS 2000, Operation Manual, Leco, St. Joseph, MI, USA) was used to load porcelain-weighted samples into the combustion chamber (1300 °C). A thermal conductivity cell was used to measure the N_2_ content.

Fe, Zn, B, and Cu concentrations were measured by atomic emission spectrometry and Inductive Coupled Plasma Optical Emission Spectrometric (ICP-OES) (Optima 4300 DV, ICP-OES, PerkinElmer Inc., Waltham, MA, USA). The ICP-OES is a collective device used to analyze all key plant components in a matter of seconds. Each element was measured at a specific emission wavelength that was chosen for its high sensitivity and lack of spectral interference. For all other elements studied, the wavelengths were 383.826 nm and 422.673 nm [42].

Finely milled samples of 0.8 g were agitated in 40 mL of 0.5 N hydrochloric acid for five minutes using a reciprocating shaker to quantify calcium. The flow rates of air and acetylene were tuned to minimize the (rich, slightly yellow) flame. Dissolving calcium carbonate in hydrochloric acid yielded a calcium standard stock solution. To avoid interferences from P and Al in the sample, a final concentration of 1% lanthanum was utilized [43].

The magnesium concentration was evaluated by weighing the dried sample of 0.8 g after drying at 105 °C and wet-combusting in a mixture of nitric acid and perchloric acid using an ICP emission spectrometer (PerkinElmer Optima 7300, Waltham, MA, USA).

### 4.5. Statistical Analysis

Data were analyzed and populated using SAS software 9.4. Variance and principal component analyses with mean separation tests were performed. An LSD test determined differences between variants, and statistical differences between treatment means were determined at *p* < 0.05 [44].

## 5. Conclusions

This study found that terpenoids were present in moderate amounts in the WE, HO, and RO genotypes, while terpenoids were slightly present in MH. Steroids were highly present in genotypes collected from MH and RO, while the WE genotypes showed moderate levels of steroids, and the HO genotypes had slightly present levels. *A. afra*’s high concentration of phenols and flavonoids are perceived to be responsible for its medicinal properties and antioxidant activity. Saponins were found in small amounts in all the genotypes collected from locations in Lesotho and South Africa. This study also found that P, Ca, K, Fe, and Zn had a positive correlation with the first PCA, while Cu had a positive correlation with both the first and second PCAs, with variabilities of 29.95% and 21.12%, respectively. The MH and HP genotypes had relatively high levels of ascorbic acid, phenolic compounds, flavonoids, and tannins. Interestingly, this study also found that the MH and HP genotypes had low levels of foliar Mg. Based on these results, this study recommends further research into the causes of phytochemical (ascorbic, flavonoid, phenolic, and tannin) variations in *A. afra* in the WP and RO geolocations, as well as the phytochemical effects of Mg. Additionally, this study suggests including more locations within the Saharan region in future studies. The MH and HP genotypes are also recommended for their high phytochemical contents.

## Figures and Tables

**Table 1:** Phytochemical assay of *A. afra* genotypes collected from various locations.

Phytochemicals	Wepener					Hobhouse					Mohale’s Hoek					Roma				
	1	2	3	4	5	1	2	3	4	5	1	2	3	4	5	1	2	3	4	5
Flavonoids																				
NaOH test	+	−	−	−	−	+	+	+	−	+	−	−	+	−	−	−	−	−	−	−
FeCl_3_ test	−	++	++	++	+	+	+	+	−	+	++	++	+	+++	+	++	++	+++	+	+++
Tannins	++	++	++	++	+	++	+	−	+	+	++	++	++	+++	+	++	++	+++	++	+++
Phenolics	+	+++	+++	+++	+++	+++	+++	++	+	+++	++	++	+++	+++	++	++	+++	++	++	+++
Terpenoids	++	++	++	+++	++	++	+++	++	−	++	+	++	+	+	−	+	+	+++	+	++
Steroids	++	++	++	++	+	+	++	+	++	+	++	+++	++	+++	++	++	+++	+++	++	+++
Saponins	−	+	+	+	+	+	++	+	−	+	+	+++	+	−	+	+	+	−	−	+
Alkaloids																				
Wagner’s test	++	+	+	+	+++	+	+	+	++	+	+++	−	+	++	++	+	+	+	++	++

**Table 2:** Foliar mineral analysis of *A. afra* collected at different locations in Lesotho (Mohale’s Hoek, 30.1426° S, 27.4674° E, and Roma, 29.5212° S, 27.7755° E) and South Africa (Wepener, 29.7294° S, 27.0206° E, and Hobhouse, 29.5258° S, 27.1452° E).

Foliar Minerals (mg kg^−1^)					
Location	N%	P	Ca	Mg	K
Hobhouse	0.39 ± 0.07 ^bc^	1.92 ± 0.16 ^bc^	17.27 ± 0.37 ^a^	6.52 ± 1.36 ^a^	1289.00 ± 102.47 ^a^
Mohale’s Hoek	0.56 ± 0.12 ^a^	2.09 ± 0.24 ^ab^	16.26 ± 2.46 ^a^	4.66 ± 0.51 ^a^	1346.93 ± 145.60 ^a^
Roma	0.43 ± 0.08 ^ab^	1.72 ± 0.06 ^c^	10.20 ± 1.30 ^b^	10.37 ± 1.98 ^b^	1166.87 ± 165.04 ^a^
Wepener	0.30 ± 0.02 ^c^	2.27 ± 0.26 ^a^	14.67 ± 2.84 ^a^	10.81 ± 1.64 ^b^	1278.25 ± 55.49 ^a^
LSD_T_ 0.05	0.12	0.31	2.96	2.48	223.3
*p*-value	**	*	***	***	ns
	Na	Fe	Cu	Zn	Mn
Hobhouse	109.14 ± 6.05 ^a^	102.60 ± 114.3 ^a^	3.68 ± 2.31 ^a^	35.43 ± 6.97 ^a^	15.25 ± 1.78 ^b^
Mohale’s Hoek	103.68 ± 7.02 ^a^	79.75 ± 60.66 ^a^	9.02 ± 4.15 ^a^	36.05 ± 5.18 ^a^	26.93 ± 7.35 ^a^
Roma	105.69 ± 3.49 ^a^	178.78 ± 38.69 ^a^	5.33 ± 1.16 ^a^	32.60 ± 4.97 ^a^	21.75 ± 3.23 ^ab^
Wepener	104.21 ± 10.02 ^a^	110.38 ± 121.49 ^a^	5.77 ± 3.19 ^a^	32.25 ± 4.28 ^a^	24.15 ± 2.96 ^a^
LSD_T_ 0.05	11.44	154.73	3.89	8.82	6.65
*p*-value	ns	ns	ns	ns	*

Degrees of freedom = 8. Statistical significance was at the 0.05 *p*-value. *** *p* < 0.0001, ** *p* < 0.001, * *p* < 0.05, ns = not significant at *p* > 0.05, ± = standard deviation, LSD_T_ 0.05 = least significant difference at the 5% level of significance. Means with the same letter as superscripts within a column are not significantly different (*p* < 0.05).

**Table 3:** Soil chemical analysis of the Lesotho region (Mohale’s Hoek, 30.1426° S, 27.4674° E, and Roma, 29.5212° S, 27.7755° E) and South Africa (Wepener, 29.7294° S, 27.0206° E, and Hobhouse, 29.5258° S, 27.1452° E).

Location	P	K	Ca	Na	Mg	Cu	Zn	Fe	Mn	Available N	pH	*OC%	**CEC
mg kg^−1^													
Wepener	3.4	100	4597	50.04	1130	1.3	3.7	36.5	7.49	112	5.37	8.95	34.12
Roma	2.7	332	6410	45.46	318	1.2	8.1	34.4	5.13	112	6.86	1.59	31.83
Mohale’s Hoek	2.1	212	8665	54.28	663	1.6	0.9	13.8	6.52	84	5.45	4.77	35.56
Hobhouse	2.2	354	895	49.30	582	1.2	5.8	18.7	4.52	140	7.08	10.74	32.75

*OC, organic carbon; **CEC, cation exchange capacity.

## Data Availability

The data are contained within the article.
